# IL-33 reduces tumor growth in models of colorectal cancer with the help of eosinophils

**DOI:** 10.1080/2162402X.2020.1776059

**Published:** 2020-06-16

**Authors:** Melanie Kienzl, Carina Hasenoehrl, Paulina Valadez-Cosmes, Kathrin Maitz, Arailym Sarsembayeva, Eva Sturm, Akos Heinemann, Julia Kargl, Rudolf Schicho

**Affiliations:** a Otto Loewi Research Center, Division of Pharmacology, Medical University of Graz, Graz, Austria; b BioTechMed, Graz, Austria

**Keywords:** Eosinophils, colorectal cancer, interleukin-33, adoptive transfer, CT26

## Abstract

In many types of cancer, presence of eosinophils in tumors correlate with an improved disease outcome. In line with this, activated eosinophils have been shown to reduce tumor growth in colorectal cancer (CRC). Interleukin (IL)-33 has recently emerged as a cytokine that is able to inhibit the development of tumors through eosinophils and other cells of the tumor microenvironment thereby positively influencing disease progress. Here, we asked whether eosinophils are involved in the effects of IL-33 on tumor growth in CRC.

In models of CT26 cell engraftment and colitis-associated CRC, tumor growth was reduced after IL-33 treatment. The growth reduction was absent in eosinophil-deficient ΔdblGATA-1 mice but was restored by adoptive transfer of *ex vivo*-activated eosinophils indicating that the antitumor effect of IL-33 depends on the presence of eosinophils. *In vitro*, IL-33 increased the expression of markers of activation and homing in eosinophils, such as CD11b and Siglec-F, and the degranulation markers CD63 and CD107a. Increased expression of Siglec-F, CD11b and CD107a was also seen *in vivo* in eosinophils after IL-33 treatment. Viability and cytotoxic potential of eosinophils and their migration properties toward CCL24 were enhanced indicating direct effects of IL-33 on eosinophils. IL-33 treatment led to increased levels of IL-5 and CCL24 in tumors.

Our data show that the presence of eosinophils is mandatory for IL-33-induced tumor reduction in models of CRC and that the mechanisms include eosinophil recruitment, activation and degranulation. Our findings also emphasize the potential use of IL-33 as an adjuvants in CRC immunotherapy.

**Abbreviations:**

AOM: azoxymethane; bmRPMI: bone marrow RPMI; CRC: colorectal cancer; CFSE: carboxyfluorescein succinimidyl ester; DSS: dextran sulfate sodium; EPX: eosinophil peroxidase; INF-γ: interferon gamma; ILC: innate lymphoid cell; IL-33: interleukin-33; IL-5: interleukin-5; MDSC: myeloid derived suppressor cells; NK cells: natural killer cells; P/S: penicillin/streptomycin; rm: recombinant mouse; T regs: regulatory T cells; TATE: tumor associated tissue eosinophilia; TNF-α: tumor necrosis factor alpha

## Introduction

Eosinophils have been originally regarded as effector cells in allergic diseases and as a defense against helminths. Over the years, however, presence of eosinophils has been consistently reported in tumor tissue (TATE, tumor associated tissue eosinophilia). Many of these reports demonstrate that tissue eosinophilia correlate with a better disease prognosis.^1^ Also in studies of human colorectal cancer (CRC), increased numbers of eosinophils in tumors have been shown to serve as an independent favorable factor correlating with better prognosis and longer patient survival.^2–5^ High counts of eosinophils, particularly in tumor stroma, were associated with a decreased risk for CRC death.^6^

A recent study has revealed that activated eosinophils play an important part in the reduction of tumor growth of intestinal cancer indicating that the presence of eosinophils in cancers of the gastrointestinal tract may oppose neoplasia.^7^ On the other hand, in a model of oral carcinogenesis, eosinophil-deficient ΔdblGATA-1 mice were less affected by tumor growth than the wild types,^8^ suggesting that the pro-/anti-tumorigenic role of eosinophils in tumor development is dependent on the type of cancer (recently reviewed in^9^). Eosinophils are able to destroy cancer cells through degranulation and the release of cytotoxic granules which contain major basic protein, eosinophil cationic protein, and eosinophil peroxidase (EPX).^1,10^ Granzyme A and tumor necrosis factor alpha (TNF-α) have been also identified as eosinophil-derived tumoricidal mediators causing apoptosis and necrosis of Colo-205 colon carcinoma cells, a process that requires CD11a/CD18-dependent adhesion of eosinophils to cancer cells.^10^ In general, eosinophils have been described as effector cells of immune surveillance that release cytokines to modulate functions of other leukocytes while also expressing receptors for cytokines, chemokines, growth factors, lipids and adhesion molecules to integrate inflammatory signals.^11^ As to their role in solid tumors, eosinophils are thought to orchestrate a program to reduce tumor growth with the help of other leukocytes such as CD8^+^ T cells that are attracted to the tumor site via eosinophil-produced chemokines.^12^

A newly emerged cytokine that is able to influence tumor development via eosinophil activation is interleukin (IL)-33.^13^ Although thought to be mostly pro-tumorigenic in many types of cancer^14-16^ including CRC,^17–24^ IL-33 was recently shown to reduce tumor growth in skin cancer and interestingly also in CRC models.^25–27^ IL-33 is a member of the IL-1 family and normally released from damaged epithelial and endothelial barrier cells acting as an alarmin.^14^ In this function, it can activate leukocytes, such as mast cells, eosinophils, Th2, regulatory T (Treg), CD8^+^ and natural killer (NK) cells, and also innate lymphoid cells (e.g. ILC2).^14^ IL-33 can influence immune cells of the tumor microenvironment by inducing IL-12-dependent Th1 cell differentiation in human and mouse CD4^+^ T cells,^28^ a cell type known to be associated with a good prognosis in CRC.^29^ In fact, IL-33 has been proposed as a vaccine adjuvants in cancer immunotherapy because it can cause antigen-specific polyfunctional CD4^+^ and CD8^+^ T-cell responses.^30^ The addition of a danger signal (alarmin) like IL-33 may help to overcome tumor immune tolerance, a major obstacle in cancer immunotherapy.^31^ On the other hand, transgenic mice expressing IL-33 in intestinal epithelial cells and crossed with APC^min/+^ mice showed increased tumor growth and higher expansion of Tregs than their littermates.^19^ It has been, therefore, suggested that IL-33 expressed in tumor cells may promote immune responses in CD8^+^ T cells and NK cells while IL-33 from tumor stroma favors tumor growth via immune suppression through Tregs and MDSCs,^31^ important findings that need to be considered before using IL-33 in immunotherapy. Recent reviews on the role of IL-33/ST2 (IL-33 receptor) in tumorigenesis have discussed in more detail how remodeling of the tumor microenvironment by IL-33 may either promote or reduce tumor growth.^16,32^

Two reports have now shown that IL-33 directly activates eosinophils and reduces pulmonary metastasis and growth of melanoma.^13,25^ As for CRC however, it is still unclear whether eosinophils play a role in IL-33-induced reduction of tumor growth. In the current study, we, therefore, used two *in vivo* models to investigate whether eosinophils are involved in the effects of IL-33 on tumor growth in CRC. We can report that IL-33 is a potent cytokine that diminishes tumor growth in both models. Using ΔdblGATA-1 mice, we further show that the effects of IL-33-induced tumor reduction are dependent on the presence of eosinophils.

## Methods

### Mice and cell line

All animal experiments were performed in the animal facilities of the Medical University of Graz. Experimental protocols were approved by the Austrian Federal Ministry of Science and Research (animal license numbers: BMWF-66.010/0076-V/3b/2018 and BMBWF-66.010/0041-V/3b/2018. BALB/c mice were either bred in house or obtained from Charles River). ΔdblGATA-1 mice were initially obtained from Dr. Helene Rosenberg (NIH, Bethesda, MD, USA) and bred in our facilities. Male CD-1 mice were obtained from Charles River. CByJ.B6-Tg(UBC-GFP)30Scha/J mice were purchased from Jackson Laboratory and bred in our facilities.

The colon carcinoma cell line CT26 was obtained from ATCC (ATCC® CRL-2638™) and maintained in RPMI with 10% FBS (Life Technologies; # 21875–091 and # 10270106) and 1% penicillin/streptomycin (P/S; PAA Laboratories; P06-07100) at 37°C and 5% CO_2_ in a humidified atmosphere.

### Murine tumor models and IL-33 treatment

For the heterotopic CRC tumor engraftment model, 1 × 10^5^ CT26 cells were injected subcutaneously (s.c.) into the flank of 8–12 week old BALB/c or ΔdblGATA-1 mice. When tumors were palpable (after ~1 week), mice were treated intraperitoneally (i.p.) with 0.4 µg recombinant mouse (rm)IL-33 (Biolegend; # 580506) or PBS (as a negative control) every other day six times in total.^25^ Tumor progression was monitored during the course of the experiment. Mice were sacrificed 24 hrs after the last injection, and tumors were collected. Tumors were weighed, and then measured with a caliper. The tumor volume was calculated by using the formula (v = length x width x height x π/6).

Colitis-associated CRC was induced in CD-1 mice as described before.^33^ In brief, mice were first injected i.p. with 10 mg/kg of azoxymethane (AOM; Sigma-Aldrich; A5486). 2% of dextran sulfate sodium (DSS; MP Biomedicals; # 216011090) was then added to their drinking water from day 7–13 and 28–34. IL-33 treatment was started seven days after the last bout of DSS and applied i.p. at 1 µg/mouse two to four times per week. On day 99, mice were sacrificed, the colon was removed and opened longitudinally. Tumors were counted and tumor areas were measured with a caliper. Tumors were excised and kept in RPMI on ice until further use (or fixed in 10% Roti®-Histofix; Carl Roth; A146.5).

### Single cell suspensions

Single cell suspensions of s.c. tumors were prepared as previously described.^34^ Tumors were cut in small pieces and digested with collagenase (CLS-1; 4.5 U/ml; Worthington) and DNase I (160 mU/ml; Worthington; LS002006) for 25 min at 37°C while rotating at 1000 rpm. Digestion was only interrupted once by shortly vortexing samples. Thereafter, tissue was passed through a 40 µm strainer and washed with PBS + 2% FBS.

Dissected tumors of the colon from the AOM+DSS model were cut in small pieces and digested in PBS (containing Ca^++^ and Mg^++^), supplemented with 5% FBS, 1 mg/ml collagenase A (Roche; #10103586001) and 160 mU/ml DNase I for 40 min at 37°C while shaking at 1000 rpm. Tissue was afterward passed through a 100 µm strainer and washed with PBS + 2% FBS. After washing, cells were counted and used for antibody staining.^35^

### Flow cytometric phenotyping of immune cell populations

Prior to immunostaining,^34^ single cell suspensions were incubated with 1 µg TruStain FcX™ (Biolegend; # 156604). Staining was then performed for 30 min on ice (protected from light) with the following antibodies: CD45-AF700 (# 103128), CD45-BV785 (# 103149), Ly6 C-APC (# 128015), Ly6 G-PE/Dazzle594 (# 127648), CD11 c-BV605 (# 117334), CD8-PerCPCy5.5 (# 100734), CD63-PE (# 143903), CD107a-BV421 (# 121618) (all antibodies from Biolegend), CD11b-BUV737 (# 612801), CD11b-PECy7 (# 561098), F4/80-BUV395 (# 565614), CD3-BUV395 (# 563565), CD4-BUV496 (# 564667), Siglec-F-PE (# 562068) (all antibodies from BD Biosciences) and FoxP3-PE (eBioscience; # 12–5773-82). For nuclear antigen staining, cells were permeabilized with Transcription Factor Buffer Set (BD Biosciences, # 562574) prior to staining procedures. Dead cells were excluded with Fixable Viability Dye (FVD) eFluor™ 780 (eBioscience; #65-0865-14) according to the manufacturer’s protocol. Stained cells were washed, fixed with IC Fixation Buffer (eBioscience; # 00–8222-49), and stored at 4°C until analysis. Samples were analyzed on a BD LSRFortessa™ flow cytometer with BD FACSDiva software (BD Biosciences, Franklin Lakes, NJ, USA). Per sample, >2 x 10^5^ events were recorded. Data were compensated and analyzed with FlowJo software (TreeStar, Ashland, OR, USA). Gates were defined by fluorescence-minus-one samples. See *Supplementary* Fig. 1 for gating strategies.

### Immunohistochemistry/histochemical staining

Paraffin-embedded sections of mouse tumors were cut (5 µm) and deparaffinized. For immunohistochemistry, sections were microwaved for 2 x 5-min cycles in 10 mM citrate buffer, and processed by ABC method according to the manufacturer’s protocol (Vectastain ABC kit; Vector Labs; PK-6101). Sections were then incubated with biotinylated mouse anti-EPX antibody (clone MM25-82.2.1; 5 µg/ml; antibody kindly donated by Dr. Elizabeth Jacobsen) and visualized with 3–3´-diaminobenzidine (DAB; Vector Labs; SK-4100). Images were taken with a high resolution digital camera (Olympus UC90) and analyzed by Olympus cellSense Standard 1.17 imaging software (Olympus, Vienna, Austria). Contrast, brightness and color balance of images were adjusted using Corel Photo Paint® (Corel Corp.). For histochemical staining of eosinophils, Sirius Red (Direct Red 80®, Sigma-Aldrich; # 365548) was used in deparaffinized sections. Sirius Red-stained tissue was counterstained with Gill’s hematoxylin II (Carl Roth; T864.2).

### Protein extraction and cytokine analysis

Snap frozen tumor tissue was lysed in RIPA buffer (Thermo Fisher; # 89900) and homogenized in a Percellys 24 homogenizer (VWR, Vienna, Austria) by using ceramic beads (VWR; #432-0356). Subsequently, the protein lysate was centrifuged at 14,000 rpm for 10 min (4°C) before protein concentration was determined by a Pierce^TM^ BCA Protein Assay Kit (Thermo Fisher; # 23227) according to the manufacturer’s protocol. Cytokine expression was evaluated by ProcartaPlex Multiplex Immunoassay (affymetrix eBioscience; PPX-13). The CCL24 ELISA was performed according the manufacturer’s protocol (Thermo Fisher; EMCCL24).

### Differentiation and activation of bone marrow derived eosinophils

Bone marrow was isolated from BALB/c and CByJ.B6-Tg(UBC-GFP)30Scha/J mice and eosinophils were differentiated as previously published.^36^ In brief, erythrocytes in bone marrow were lysed using ddH_2_O followed by neutralization with 10xPBS. The cells were cultured in bmRPMI, i.e. RPMI + 20% HyClone FBS (GE Healthcare; # 10309433), 1% P/S, 25 mM HEPES (Thermo Fisher; # 15630–080), 1 x non-essential amino acids (Thermo Fisher; # 11140–035), 1 mM sodium pyruvate (Thermo Fisher; # 11360–039) and 50 µM beta-mercaptoethanol (Sigma-Aldrich; M3148) supplemented with 100 ng/ml stem cell factor (PreproTech; # 250–03) and 100 ng/ml FLT3L (PreproTech; # 250–31 L). On day four, medium was changed to bmRPMI supplemented with 10 ng/ml IL-5 (Bio-Techne; # 405-Ml-005) only, to differentiate progenitors into eosinophils. Fresh medium was added every second day. On day 8 and 12, cells were transferred into a new flask. On day 13, 100 ng/ml IL-33 was added to half of the eosinophils for activation (referred to as IL-33 Eos) while the other half was kept in bmRPMI/IL-5 alone which served as control (referred to as IL-5 Eos). Before use, eosinophils were washed twice in PBS to remove IL-33 and IL-5. Purity and viability was checked using flow cytometry. For intravenous (i.v.) injections, IL-5 Eos and IL-33 Eos were either stained with the fluorescent markers CFSE or eFluor^TM^ 450 (eBioscience; #65-0842-85 and #65-0820-84) according to the manufacturer’s protocol) or used unstained, at a concentration of 5–10 × 10^6^ cells/200 µl PBS per recipient mouse.

### Eosinophil migration assay

Eosinophil migration assays were performed using 5 µm trans-well plates (Corning; CLS3387-8EA) as previously described by us.^37^ In brief, 1 × 10^5^ IL-33 Eos (or IL-5 Eos) were put in the upper well. Supernatant from CT26 cell lines (conditioned for four days) and unconditioned medium was used for chemoattraction in the lower well. Recombinant CCL24 (eotaxin-2; Immunotools; # 11344174) was used as a positive control for eosinophil migration at the indicated concentrations. Eosinophils that migrated to the lower well were enumerated on a FACS Canto (BD Biosciences) as described previously.^38^

### Cytotoxicity and viability assay

Eosinophil cytotoxicity assays were carried out as described before.^7^ IL-5 Eos (or IL-33 Eos) were co-incubated with CT26 cells (4x10^4^) in a 96-well plate at different ratios for eosinophils to tumor cells (E:T). To be able to differentiate between eosinophils and CT26 cells, we either used GFP^+^ eosinophils from CByJ.B6-Tg(UBC-GFP)30Scha/J mice (Jackson Laboratory) or we stained CT26 cells with eFluor^TM^ 450 (500 nM). After 6, 7 or 24 hrs, eosinophils were collected, and CT26 tumor cells were detached using trypsin/EDTA (PAN Biotech; P10-023100). Cells were stained with Annexin-V (BD Biosciences; # 5566547; according to the manufacturer’s protocol) or Zombie NIR^TM^ Fixable viability dye (Biolegend; # 423105). The percentage of dead CT26 cells (or living GFP^+^ eosinophils) was then analyzed as Annexin-V or Zombie NIR^TM^ Fixable viability dye positive or negative cells using flow cytometry.

### Statistical analysis

Statistical analyses for *in vitro* and *in vivo* experiments was performed using GraphPad Prism 6.1 (GraphPad Software). Significant differences between two experimental groups were determined using unpaired or paired student’s *t-*tests, multiple *t*-tests or two-way ANOVA with the Sidak’s post hoc test for corrections of multiple comparisons. For comparison of three groups, one-way ANOVA was used with the indicated post hoc test for corrections of multiple comparisons.

## Results

### IL-33 increases infiltration of eosinophils into tumors of CRC models and causes reduction of tumor growth

**Figure 1.**
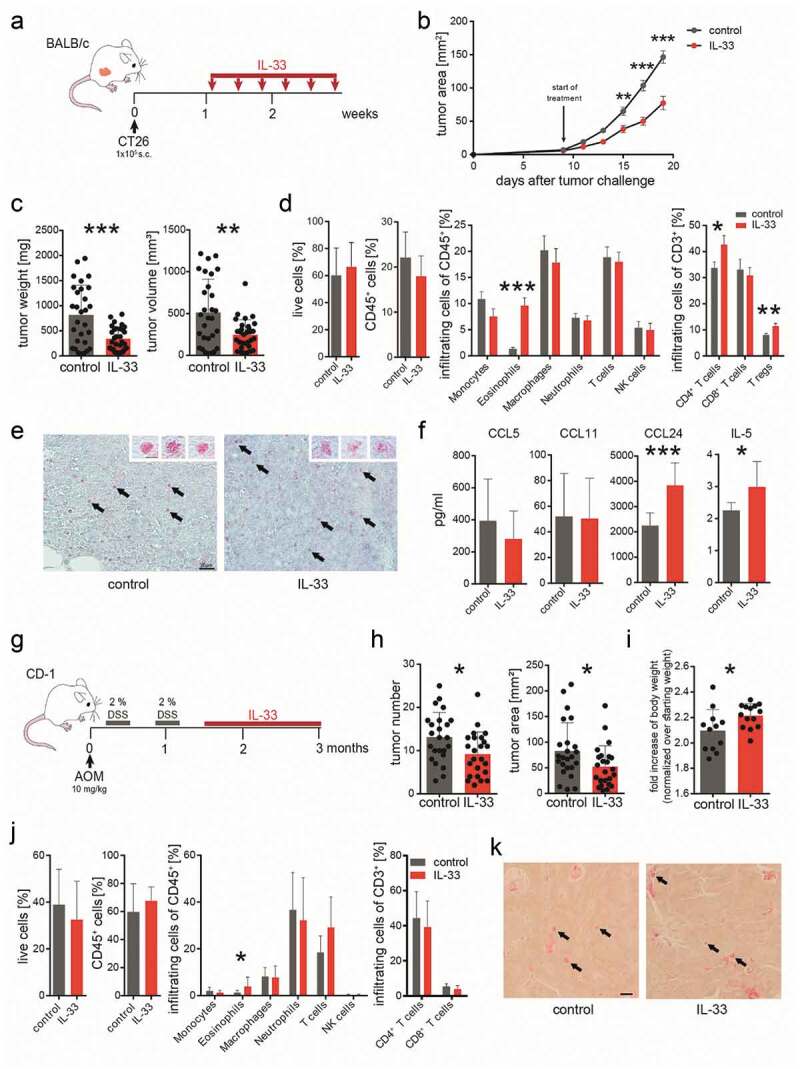
IL-33 causes eosinophil infiltration into tumors and reduced tumor growth in models of CRC.

In the CT26 cell engraftment tumor model with BALB/c mice (Figure 1a), tumor growth was decelerated by treatment with IL-33 as compared to vehicle treatment (control) (Figure 1b). Weights and volumes of tumors, measured at the end of the experiment, were reduced by more than 50% (Figure 1c). Flow cytometric analysis of tumors revealed increased infiltration of eosinophils, CD4^+^ T cells and Tregs after IL-33 vs. vehicle treatment (control) (Figure 1d). In Sirius Red stainings from tumors of IL-33-treated mice, eosinophils appeared less granulated than those from tumors of vehicle-treated (control) animals (inserts at the right upper corners of the images) suggesting that degranulation of eosinophils has occurred *in vivo* (Figure 1e, also see *Supplementary* Figure 2 showing anti-EPX antibody-stained eosinophils). We also detected increased levels of IL-5 and CCL24 in tumors of IL-33-treated mice, whereas levels of CCL11 (eotaxin-1) and CCL5 did not differ between the two groups (figure 1f).

(a) BALB/c mice were subcutaneously (s.c.) engrafted with 1 × 10^5^ CT26 colon cancer cells (time point 0). IL-33 treatment (0.4 µg/mouse i.p. every other day for six times) was started when tumors were palpable (after ~1 week). (b) Tumor development was monitored during the course of the treatment. Data indicate mean values ± SEM from 3 independent experiments. n = 23–25. (c) One day after the last IL-33 injection, mice were sacrificed and tumor weight and volume was measured *ex vivo*. Data were pooled from three independent experiments. n ≥ 29. (d) Flow cytometric analysis of single cell suspensions from s.c. tumors. Data were pooled from 3 independent experiments; n = 13–21. (e) Sirius Red staining of eosinophils in s.c. tumors of IL-33- and vehicle-treated (control) mice (representative images from n = 3/group; calibration bar: 20 µm). Arrows denote examples of Sirius Red-stained eosinophils. Inserts in the right upper corners show enlarged Sirius Red-stained eosinophils with granules easily detectable (calibration bar: 5 µm). (f) Immunoassay of lysed tumor tissue shows significant differences in the levels of IL-5 and CCL24 between IL-33- and vehicle-treated (control) mice; n = 7–10. Data indicate mean values ± SD. (g) Schematic presentation of the AOM+DSS-induced CRC model as performed in CD-1 mice treated with 1 µg IL-33/mouse i.p. 2–4 times per week. (h) Tumor number and area were measured in each colon showing a reduction in tumor growth in the AOM+DSS+IL-33 treated mice; n = 24–27. (i) Body weights (normalized to the starting weight) of IL-33- and vehicle-treated (control) mice. Data represent one of two independent experiments; n = 12–14. (j) Flow cytometric analysis of tumor infiltrated leukocytes. Data indicate mean values ± SD; n = 7–18. (k) Sirius Red staining of eosinophils in tumor tissue of AOM+DSS+IL-33- and vehicle-treated (control) mice (calibration bar: 20 µm; representative image from n = 3–4/group). Arrows denote representative examples of Sirius Red-stained eosinophils. Statistical differences were assessed by using two-way ANOVA with Sidak’s post hoc test and unpaired student’s *t*-test. *p < .05; **p < .01; ***p < .001.

In the AOM+DSS colitis-associated CRC model (Figure 1g), IL-33 was applied one week after the last cycle of DSS in order not to interfere with the (inflammatory) effect of DSS. Also here, treatment with IL-33 reduced tumor growth, as evaluated by tumor number and tumor area (Figure 1h). By the end of the experiment, IL-33-treated animals had also gained more weight than the ones treated with vehicle (control) (see fold bodyweight in Figure 1i). Our flow cytometric data showed that the number of eosinophils was significantly increased in tumors by IL-33 treatment (Figure 1j). We did not detect differences in the other investigated leukocytes between the two groups (Figure 1j). Sirius Red stainings of eosinophils in colonic tumors of the AOM+DSS colitis-associated CRC model are shown in Figure 1k. Eosinophils can be seen throughout the tumor tissue, typically located in adenomatous crypts.

### IL-33 increases markers of activation, homing and degranulation in eosinophils

**Figure 2.**
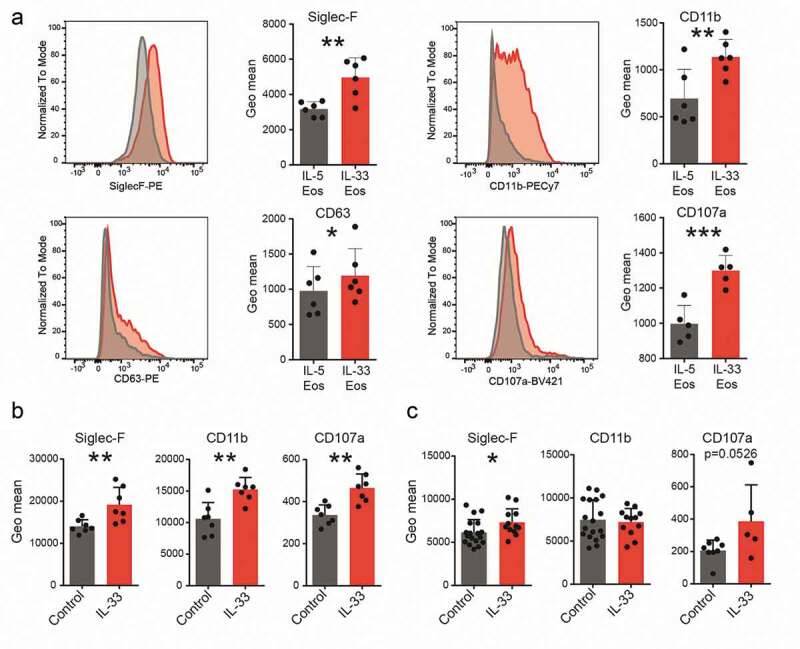
Expression of markers for activation, homing and degranulation.

To investigate whether IL-33 causes anti-tumorigenic effects directly via eosinophils, we first differentiated bone marrow eosinophils from BALB/c mice and added 100 ng/ml of IL-33 for 20 hrs to the culture medium (IL-33 Eos). The other half of the cells was kept in normal IL-5-supplemented culture medium (IL-5 Eos) and served as a control. *In vitro*, incubation with IL-33 increased the expression of markers for activation and homing such as CD11b^13,39^ and Siglec-F,^40^ and markers of degranulation, such as CD63^13,41^ and CD107a,^42^ in eosinophils (Figure 2a).

Bone marrow-derived eosinophils were activated with IL-33 (IL-33 Eos) for 20 hrs or incubated with IL-5 (IL-5 Eos) as a control, and marker expression was evaluated using flow cytometry. (a) Histograms show representative flow cytometry experiments. Next to the histograms, the graphs show paired data of two treatments (IL-5 and IL-33). Data represent one of two independent experiments; n ≥ 5. (b) Expression of markers for homing and activation (Siglec-F and CD11b) and for degranulation (CD107a) were evaluated in tumor infiltrating eosinophils from IL-33- and vehicle-treated BALB/c mice (control) (s.c. tumor model; n = 7) and from (c) IL-33- and vehicle-treated (control) AOM+DSS mice (data pooled from two independent experiments; n = 12–18. Statistical differences were assessed by using paired and unpaired student’s *t*-test. *p < .05; **p < .01; ***p < .001.

Eosinophils were also investigated in tumors of the *in vivo* models. In s.c. engrafted mice, eosinophils showed increased expression of Siglec-F, CD11b and CD107a post IL-33 treatment (Figure 2b). In eosinophils from tumors of IL-33-treated AOM+DSS mice, Siglec-F but not CD107a (*p* = .0526) was significantly higher than in vehicle-treated (control) animals. No change for CD11b was detected in the AOM+DSS+IL-33 mice vs. vehicle (control) (Figure 2c). Our data, therefore, indicate that IL-33 leads to increased expression of molecules involved in activation, homing and degranulation of eosinophils *in vitro* and *in vivo*.

### IL-33 enhances migration and viability properties of eosinophils

**Figure 3.**
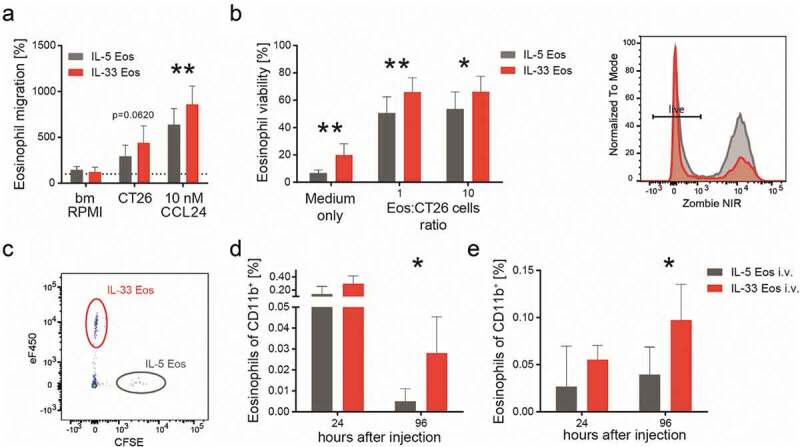
IL-33 dependent differences in migration and survival of eosinophils.

Because infiltration of eosinophils into tumors was increased in response to IL-33 treatment we next investigated whether migration of eosinophils toward CT26 cell-conditioned supernatant and CCL24 could be modulated by IL-33 pre-treatment. By use of trans-well migration assays, we observed that activation with IL-33 enhanced the migration of bone marrow-derived eosinophils toward CCL24 as compared to IL-5 Eos, and also slightly (though not significantly; *p* = .062) toward CT26 cell-conditioned medium (Figure 3a).

(a) The migration of eosinophils (pre-activated with IL-33 [IL-33 Eos] or incubated with IL-5 alone [IL-5 Eos; serving as control]) toward CT26 cell-conditioned medium and toward CCL24 is shown. Data are means ± SD, pooled from three independent experiments (n = 7) and expressed as % of vehicle (bmRPMI). (b) Eosinophil viability was measured after 24 hrs co-incubation with indicated ratios of CT26 cells and medium only, and identified as Zombie NIR^TM^ fixable viability dye (Zombie NIR) negative cells (means ± SD, n = 3). The histogram shows a representative experiment (grey = IL-5 Eos, red = IL-33 Eos). (c) A representative flow cytometry plot shows eosinophils (IL-33 Eos and IL-5 Eos) which were injected intravenously (i.v.) into ∆dblGATA-1 mice and detected in the blood after 24 hrs. IL-33 Eos (eFluor^TM^ 450 [eF450]-stained) and IL-5 Eos (CFSE-stained) were identified as live/CD45^+^/CD11b^+^ cells. (D, E) Graphs show IL-33 Eos and IL-5 Eos (% of CD11b^+^) in the blood (d) and in subcutaneous CT26 tumors 24 and 96 hrs after adoptive transfer of eosinophils into ∆dblGATA-1 mice (e); n = 4–5. Statistical differences were assessed by two-way ANOVA with Sidak’s post hoc test, multiple *t*-tests and by unpaired student’s *t*- test.*p < .05; **p < .01.

IL-33 is also an important cytokine for eosinophil survival^43^ but whether the tumor microenvironment influences this property is unknown. We carried out *in vitro* experiments and noticed increased survival of IL-33 Eos when they were co-incubated at different ratios with CT26 cells (or medium only) indicating that CT26-conditioned medium enhanced eosinophil viability (Figure 3b). We then investigated the role of IL-33 in eosinophil migration and survival *in vivo* using eosinophil-deficient ∆dblGATA-1 mice engrafted s.c. with CT26 cells. After two weeks, we injected IL-33 Eos (eFluor^TM^ 450-stained) and IL-5 Eos (CFSE-stained) i.v. into the mice (in a 1:1 ratio/mouse) and monitored the distribution and viability of the injected eosinophils 24 and 96 hrs post-injection. A representative flow plot for the identification of the i.v. injected eosinophils, gated as live/CD45^+^/CD11b^+^ eFluor^TM^ 450^+^ (IL-33 Eos) and CFSE^+^ (IL-5 Eos), is shown in Figure 3c. Prolonged survival of IL-33 Eos (as compared to IL-5 Eos) could be detected for up to 96 hrs post eosinophil injection in the blood of ∆dblGATA-1 mice (Figure 3d). CT26 cell-engrafted tumors of ∆dblGATA-1 mice showed an increased number of IL-33 Eos after 24 hours (though not significantly different to IL-5 Eos; Figure 3e). However, significantly more IL-33 Eos (vs. IL-5 Eos) were seen 96 hrs after eosinophil injection (Figure 3e). Altogether, these findings suggest that IL-33 primes eosinophils for migration and prolonged survival not only *in vitro* but also *in vivo*.

### IL-33-induced reduction of tumor growth depends on the presence of eosinophils

**Figure 4.**
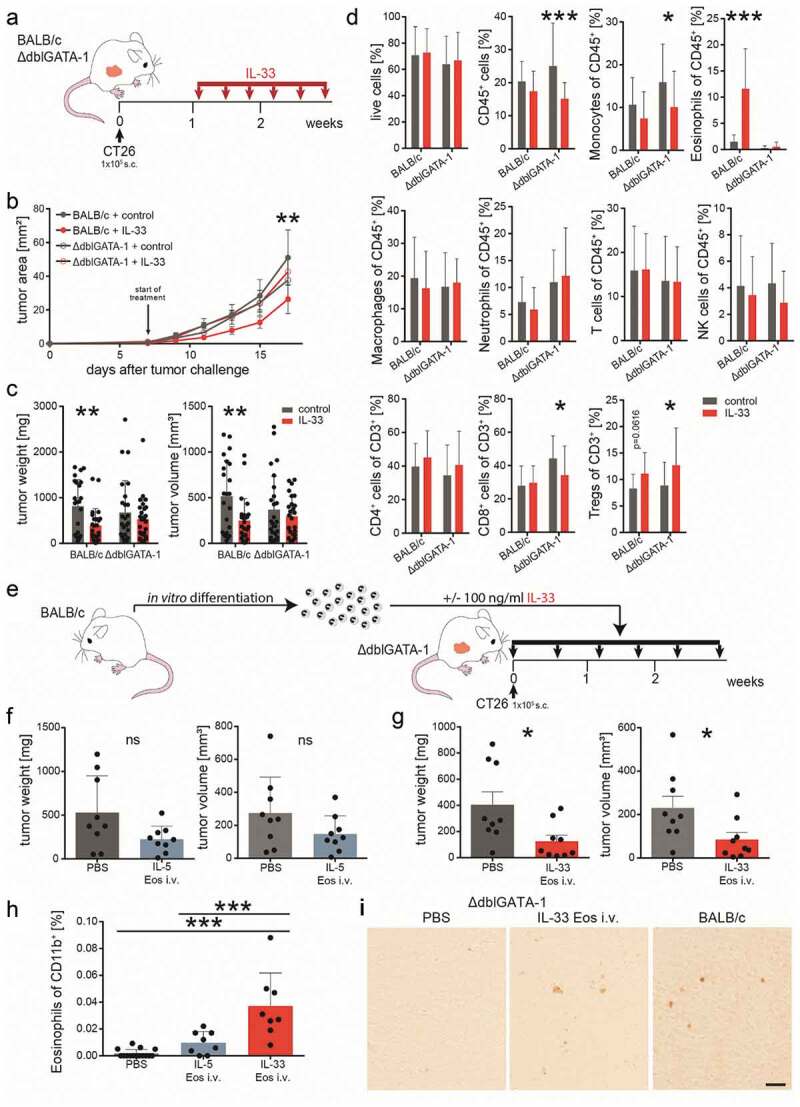
Eosinophils are necessary for a reduction in tumor growth by IL-33.

To test whether the anti-tumorigenic effect of IL-33 depends on the presence of eosinophils we used eosinophil deficient ∆dblGATA-1 mice. After s.c. engraftments with CT26 cells, mice were treated with 0.4 µg rmIL-33 every other day i.p. as described before (BALB/c mice received the same treatment) (Figure 4a). There was no difference in the time course of tumor development between IL-33- and vehicle-treated (control) ∆dblGATA-1 mice (Figure 4b). Similarly, no differences in tumor weight and volume were observed in ∆dblGATA-1 mice (Figure 4c) (we also failed to see differences in the proliferation of IL-33- and vehicle-incubated CT26 cells *in vitro*; see *Supplementary* Fig. 3), while treatment of BALB/c mice confirmed our observation that IL-33 reduces tumor growth (Figure 4b,c). Flow cytometric analysis of tumor single cell suspensions showed a reduction in CD45^+^ cells, monocytes, CD8^+^ T cells and an increase in Tregs after treatment with IL-33 vs. vehicle in ∆dblGATA-1 mice (Figure 4d). There was a marked increase of eosinophils and a slight, though not significant, increase of Tregs (*p* value =.0616) in IL-33 vs. vehicle-treated BALB/c mice (Figure 4d).

(a) Schematic presentation of the subcutaneous (s.c.) tumor model performed in BALB/c and eosinophil deficient ∆dblGATA-1 mice. IL-33 was given i.p. at a concentration of 0.4 µg/mouse every other day for a total of six times. (b) Tumor growth was monitored during the course of the experiment; n ≥ 8. Data indicate mean values ± SEM. **p < .01 BALB/c + control vs. BALB/c + IL-33; no significance between other groups. (c) One day after the last IL-33 injection, BALB/c and ∆dblGATA-1 mice were sacrificed and tumor weight and volume were measured *ex vivo* showing no differences between IL-33- and vehicle (control) treatment in the ∆dblGATA-1 but tumor growth reduction in the BALB/c mice; n ≥ 23, three independent experiments. (d) Flow cytometric analysis of single cell suspensions of tumors from BALB/c and ∆dblGATA-1 mice indicating significant differences between IL-33- and vehicle-treated (control) ∆dblGATA-1 mice for CD45^+^, monocytes, CD8^+^ T cells and Tregs, as well for eosinophils between IL-33- and vehicle-treated (control) BALB/c mice; n = 24; three independent experiments. (e) ∆dblGATA-1 mice with s.c. tumors were repopulated by adoptive transfer (twice weekly) with either IL-33 Eos or IL-5 Eos (eosinophils were isolated from bone marrow of BALB/c mice and differentiated). (f) Adoptive transfer with IL-5 Eos failed to significantly restore tumor reduction in ∆dblGATA-1 mice (in comparison to ∆dblGATA-1 mice given PBS only). (g) However, adoptive transfer with IL-33 Eos (vs. PBS only) significantly restored reduction of tumor growth; n = 8–9. (h) Single cell suspensions of the s.c. tumors were prepared and infiltrated eosinophils were evaluated as % of CD11b^+^ cells; n = 8–15. (i) Immunohistochemistry of s.c. tumors with an anti-EPX antibody shows that PBS-treated ∆dblGATA-1 mice are devoid of eosinophils. After adoptive transfer of IL-33 Eos (by i.v. injection), EPX staining is visible in the tumors of ∆dblGATA-1 mice. Infiltrated eosinophils in s.c. tumors of BALB/c mice are shown for comparison (representative images of n = 3/group; calibration bar: 20 µm). Statistical differences were assessed by using multiple *t*-tests, one-way ANOVA with Tukey’s multiple comparisons test and unpaired student’s *t*-test. *p < .05; **p < .01; ***p < .001, ns = not significant.

Our data suggest that eosinophils are needed for IL-33-dependent reduction of tumor growth.

### Activation of eosinophils by IL-33 enhances the reduction of tumor growth

To confirm that eosinophils are indispensable for the IL-33-induced reduction in tumor growth we argued that repopulation of ∆dblGATA-1 mice with eosinophils would restore the tumor growth-reducing effect. IL-33 Eos or IL-5 Eos were, therefore, injected twice a week i.v. into ∆dblGATA-1 mice bearing s.c. CT26 tumors (Figure 4e). Differentiation and viability of IL-33 Eos and IL-5 Eos were about 90% at the time of i.v. injections (see *Supplementary* Fig. 4). Tumors were slightly, though not significantly, reduced in mice repopulated with IL-5 Eos as compared to mice that did not receive eosinophils (PBS only) (Figure 4f). However, when IL-33 Eos were injected i.v. into ∆dblGATA-1 mice, tumor volumes and weights were significantly reduced (Figure 4g). The percentage of IL-33 Eos in tumors was clearly increased (vs. IL-5 Eos) (Figure 4h). Figure 4i shows immunohistochemistry of s.c. tumors in ∆dblGATA-1 mice infiltrated with IL-33 Eos.

We not only applied IL-33 pre-incubated eosinophils into CT26 tumor-bearing ∆dblGATA-1 mice i.v., but also injected IL-33 i.p. into ∆dblGATA-1 mice repopulated with IL-5 Eos and we detected reduced tumor growth (*Supplementary* Fig. 5A-B), but, unlike in Figure 4h, this time the number of tumor-infiltrated eosinophils was not significatly increased (i.p. IL-33 vs. i.p. vehicle [PBS], *Supplementary* Fig. 5 C). However, eosinophils in tumors of mice injected with IL-33 i.p. showed increased CD107a expression (vs. mice injected i.p. with PBS vehicle) indicating increased degranulation of eosinophils (*Supplementary* Fig. 5D). Collectively, the results demonstrate that IL-33 enhances the antitumor properties of eosinophils *in vivo*.

### Eosinophils activated by IL-33 have increased cytotoxic potential

**Figure 5.**
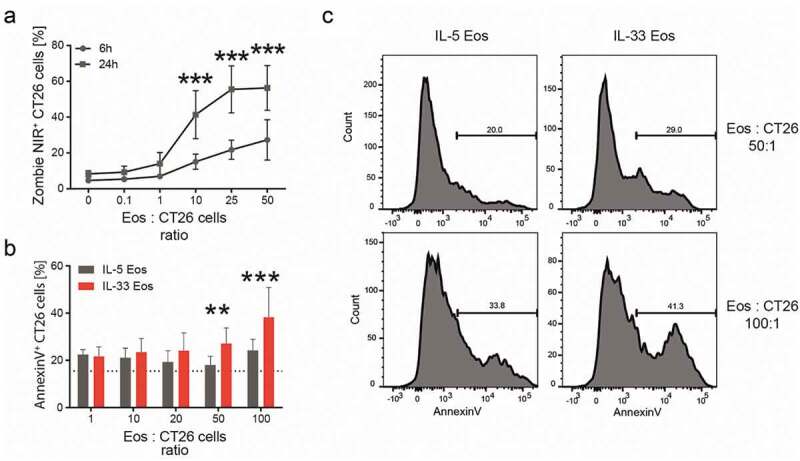
Cytotoxicity of eosinophils against CT26 cells *in vitro.*

Due to the increased expression of degranulation markers in eosinophils after IL-33 treatment *in vitro* (Figure 2a) and in tumors of BALB/c and ∆dblGATA-1 mice (adoptively transferred with eosinophils) *in vivo* (Figure 2b and *Supplementary Fig*. 5), we investigated a possible mechanism by which eosinophils could contribute to tumor growth reduction that is associated with degranulation. We, therefore, looked at the eosinophil’s potential of killing tumor cells. Eosinophil-mediated cancer cell killing was described earlier in different kinds of solid tumors, including CRC and melanoma.^7,25^ We investigated this effect also in our *in vitro* settings and found that co-incubation of eosinophils with increasing ratios of CT26 cells for 6 hrs increased the number of dead CT26 tumor cells (Figure 5a). A stronger increase in dead CT26 tumor cells was observed after a 24 hr co-incubation (Figure 5a). Since it was shown that the activation of eosinophils with IL-33 could increase death in melanoma cells^25^ we also tested this finding in our settings and co-incubated IL-33 Eos and IL-5 Eos with CT26 cells at different ratios. Compared to CT26 cells incubated with IL-5 Eos, the activation of eosinophils with IL-33 led to increased percentages of dead (Annexin-V^+^) CT26 cells (Figure 5b,c).

(a) Bone marrow-derived eosinophils (Eos) were co-incubated with CT26 cells at different eosinophil (Eos):CT26 cell ratios for 6 and 24 hrs. The percentage (%) of dead (Zombie NIR^+^) CT26 cells is depicted as mean ± SD; n = 5. (b) CT26 cells were co-incubated with either IL-5 Eos or IL-33 Eos at different Eos:CT26 cells ratios for 7 hrs (n = 5). Percentages (%) of AnnexinV^+^ CT26 cells are shown. (c) Representative histograms of IL-5 Eos and IL-33 Eos co-incubated with CT26 cells for 7 hrs at indicated ratios. Statistical differences were assessed using two-way ANOVA with Sidak’s post hoc test. **p < .01, ***p < .001.

## Discussion

IL-33 is a cytokine (alarmin) released upon necrotic cell death, particularly, by wounded epithelial and endothelial cells.^14,44^ In the context of cancer, it has been described as a pro- and anti-tumorigenic cytokine, depending on the tumor entity (reviewed in^16^). Recently, IL-33 has been linked with eosinophil activation, TATE and reduced tumor growth in a model of skin cancer.^13,25^ By using two different CRC models, a s.c. tumor cell engraftment and an AOM+DSS-driven colitis-associated model, we can show that administration of IL-33 reduces tumor growth in CRC. The reduction is dependent on the presence and the activation status of eosinophils.

Several studies have previously addressed the role of the IL-33/ST2 axis in CRC showing either tumor promoting^17-24^ or tumor reducing effects.^26,27,45,46^ Concerning the tumor microenvironment, tumor promoting effects by IL-33 have been linked with Tregs, which suppress tumor immunity,^18,19^ and with macrophage infiltration into tumors to stimulate PGE_2_ production that enhances colon cancer cell stemness.^20^ In our present work, we primarily focused on the role of eosinophils in the microenvironment and whether they might play a role in IL-33-induced reduction in tumor growth. In accordance with a report in melanoma by Lucarini et al.,^25^ we observed that IL-33 reduced the growth of tumors in mice engrafted with the colon cancer cell line CT26. The reduction was accompanied by an increased number of eosinophils and higher levels of IL-5 and CCL24 in the tumors (but not of CCL11 and CCL5) suggesting that these mediators likely contribute to increased eosinophil infiltration. Our observation that IL-33 can drive IL-5 and CCL24 expression is also supported by another study describing that in eosinophilia of airway inflammation, IL-33-stimulated production of IL-5 is accompanied by increased levels of CCL24, but not of CCL11.^47^

Despite several reports highlighting the influence of eosinophils on the nature of immune cell infiltrates in various solid tumor models,^12,25,48,49^ we observed that IL-33-induced changes in the microenvironment of the CT26 tumors only occurred in CD4^+^ T cells and Tregs but not M1 macrophages, myeloid derived suppressor cells (MDSCs), CD8^+^ T or NK cells. In accordance with other studies,^18,19^ Tregs slightly increased in CT26 tumors post-IL-33 treatment but this increase was not associated with a higher tumor burden. We also found a higher number of Tregs in tumors of ∆dblGATA-1 mice after IL-33 treatment vs. control with no change in tumor burden, suggesting that the increase in Tregs occurred independently of the presence of eosinophils. Unlike in the BALB/c mice, we also detected a decrease in CD8^+^ T cells in the microenvironment of the ∆dblGATA-1 mice post IL-33 treatment. Given the fact that eosinophils produce chemokines for the attraction of CD8^+^ T cells into the tumor microenvironment^12^ the deficiency of eosinophils may have reduced the infiltration of CD8^+^ T cells into tumors, however, it is not clear why this is only apparent after IL-33 treatment. Since IL-33 has been shown to promote tumor infiltration of CD8^+^ T cells^16^ one would have expected an increase in CD8^+^ T cells after IL-33 treatment but we did not observe this effect in the BALB/c mice. As ∆dblGATA-1 mice have been also described to show disturbed immune formation in the intestines,^50^ it is possible that less CD8^+^ T cells were formed and that IL-33 could play some role in it. Furthermore, we detected reduced infiltration of monocytes in ∆dblGATA-1 mice post IL-33 treatment which has been previously shown in wild type mice with melanoma.^25^ Considering the minor changes in the tumor microenvironment after IL-33 treatment, our data rather suggested a direct anti-tumorigenic effect of eosinophils on tumor cells. We, therefore, performed *in vitro* experiments by co-incubating eosinophils with CT26 cells and showed IL-33 dependent cytotoxic properties of eosinophils, corroborating data from studies in human and murine CRC and melanoma.^7,10,13,25^

That lymphocytes may not be needed for the effects of eosinophils on cancer growth has been previously reported in different models of heterotopically engrafted solid tumors,^51^ as well as in colitis-associated CRC models and Apc^min/+^ mice, which develop intestinal tumors spontaneously.^7^ In our AOM+DSS-induced CRC model we detected IL-33-induced TATE, which was accompanied by reduced tumor growth, but we failed to see changes in the other leukocytes measured. The role of IL-33 was also investigated in colitis-associated CRC in other reports, showing that genetic depletion of IL-33 leads to a disruption in the IgA-microbiota axis and to an increased number of tumors, but no data on the tumor microenvironment were included for the colitis-associated CRC model.^48^ On the other hand, Eissmann et al. investigated genetic ablation of the IL-33 receptor ST2 in a sporadic mouse model of CRC applying AOM over several weeks.^26^ In that study, genetic ST2 deficiency led to increased formation of colonic tumors, depending on the suppression of IFN-γ gene expression^26^ (IFN-γ expression was unaltered in our study; unpublished data). Although ST2 deficiency did not affect the infiltration of CD8^+^ T cells and myeloid cells into the tumors, it did increase the frequency of tumor-infiltrating Tregs.^26^ At last, one study shows inhibiting effects of IL-33 on colon cancer cell growth,^45^ however, we did not observe direct effects of IL-33 on CT26 cell proliferation (*Supplementary* Fig. 3). Collectively, these studies support our work on the role of IL-33 in reducing tumor growth in CRC.

IL-33 is known to lead to activation of eosinophils^52,53^ which is accompanied by upregulation of molecules important for eosinophil transmigration (CD11b) and degranulation (CD63).^13,25,39,54^ We could confirm these observations in our experiments for *in vitro-*activated eosinophils as well as for tumor-infiltrated eosinophils *in vivo*. We furthermore detected an increased expression of CD107a, which is, next to CD63, another marker of degranulation,^42^ indicating that degranulation of eosinophils most likely represents a crucial mechanism of reducing growth of tumors in our experiments. The increased expression of Siglec-F on eosinophils in tumors of IL-33-treated mice is of particular interest because high Siglec-F expression has been associated with activation and migration of eosinophils to the airways.^40^ This suggests that treatment with IL-33 may have made eosinophils more susceptible to migration and degranulation. Similarly, our migration assays with eosinophils *in vitro* demonstrated increased migration of IL-33 Eos toward CCL24 as compared to IL-5 Eos while migration toward CT26 cell-conditioned supernatant was less pronounced (*p* value .062).

Eosinophil depletion using anti-Siglec-F antibodies was used in the past to evaluate the engagement of eosinophils in tumor reduction.^12,25^ A recently published article now suggests that eosinophil depletion by anti-Siglec-F antibodies does not result in reduction of eosinophil numbers but rather in an inhibited interaction of Siglec-F with its ligands leading to the observed biological effects.^55^ Therefore, we used ∆dblGATA-1 mice in order to test whether the anti-tumorigenic effect of IL-33 treatment was dependent on the presence of eosinophils. IL-33 treatment of CT26 tumor-bearing ∆dblGATA-1 mice showed no decrease in tumor size. The repopulation of tumor-bearing ∆dblGATA-1 mice with eosinophils revealed that significantly smaller tumors developed only in mice that were injected with IL-33-activated eosinophils. In addition, more eosinophils infiltrated tumors in CT26 tumor-bearing ∆dblGATA-1 mice injected with IL-33 Eos vs. mice injected with IL-5 Eos. Our observations confirm data from a different study describing decreased growth of melanoma only when tumor cells were co-injected with IL-33-activated eosinophils.^13^

The viability of eosinophils may be significantly affected by tumor cells themselves. As recently demonstrated, the life span of eosinophils is extended independently of IL-5 when they are co-incubated with supernatant of colon cancer cells overnight.^7^ In line with this finding, we could also detect an increase in viable eosinophils when they were co-incubated with CT26 cells. It is known that IL-33 prolongs eosinophil survival^52^ and that CT26 cells produce IL-33.^27,56^ Therefore, CT26 cell-derived IL-33 in our supernatants could have been a factor to increase survival of eosinophils. Finally, we also tested whether the effects of colon cancer cell supernatant and IL-33 were additive. Our findings showed that IL-33 enhances the extended survival of eosinophils in the CRC tumor microenvironment both *in vitro* and *in vivo*.

In conclusion, our study suggests that eosinophils play an indispensable role in the tumor growth-reducing effect of IL-33 in models of CRC. Reduction in tumor growth is significantly enhanced when eosinophils are activated by IL-33. Degranulation of eosinophils seems to be a major mechanism that contributes to the IL-33 dependent anti-tumorigenic effects while the change in the tumor microenvironment may play a minor role. Our data, therefore, strongly emphasizes the importance of eosinophils and their activation when considering IL-33 as a target of therapy against CRC.

## Supplementary Material

Supplemental Material

## References

[1] Davis BP, Rothenberg ME. Eosinophils and cancer. Cancer Immunol Res. 2014;2(1):1–12. doi:10.1158/2326-6066.CIR-13-0196. 24778159

[2] Pretlow TP, Keith EF, Cryar AK, Bartolucci AA, Pitts AM, Pretlow TG, Kimball PM, Boohaker EA. Eosinophil infiltration of human colonic carcinomas as a prognostic indicator. Cancer Res. 1983;43:2997–3000. 6850611

[3] Nielsen HJ, Hansen U, Christensen IJ, Reimert CM, Brünner N, Moesgaard F. Independent prognostic value of eosinophil and mast cell infiltration in colorectal cancer tissue. J Pathol. 1999;189(4):487–495. doi:10.1002/(SICI)1096-9896(199912)189:4<487::AID-PATH484>3.0.CO;2-I. 10629548

[4] Harbaum L, Pollheimer MJ, Kornprat P, Lindtner RA, Bokemeyer C, Langner C. Peritumoral eosinophils predict recurrence in colorectal cancer. Mod Pathol. 2015;28(3):403–413. doi:10.1038/modpathol.2014.104. 25216222

[5] Fernández-Aceñero MJ, Galindo-Gallego M, Sanz J, Aljama A. Prognostic influence of tumor-associated eosinophilic infiltrate in colorectal carcinoma. Cancer. 2000;88(7):1544–1548. doi:10.1002/(SICI)1097-0142(20000401)88:7<1544::AID-CNCR7>3.0.CO;2-S. 10738211

[6] Prizment AE, Vierkant RA, Smyrk TC, Tillmans LS, Lee JJ, Sriramarao P, Nelson HH, Lynch CF, Thibodeau SN, Church TR, et al. Tumor eosinophil infiltration and improved survival of colorectal cancer patients: iowa women’s health study. Mod Pathol. 2016;29(5):516–527. doi:10.1038/modpathol.2016.42. 26916075 PMC4848192

[7] Reichman H, Itan M, Rozenberg P, Yarmolovski T, Brazowski E, Varol C, Gluck N, Shapira S, Arber N, Qimron U et al. Activated eosinophils exert antitumorigenic activities in colorectal cancer. Cancer Immunol Res. 2019;7(3):388–400. doi:10.1158/2326-6066.CIR-18-0494. 30665890

[8] da Silva JM, Queiroz-Junior CM, Batista AC, Rachid MA, Teixeira MM, da Silva TA. Eosinophil depletion protects mice from tongue squamous cell carcinoma induced by 4-nitroquinoline-1-oxide. Histol Histopathol. 2014;29(3):387–396. doi:10.14670/HH-29.387. 24105297

[9] Varricchi G, Galdiero MR, Loffredo S, Lucarini V, Marone G, Mattei F, Marone G, Schiavoni G. Eosinophils: the unsung heroes in cancer? Oncoimmunology. 2017;7(2). doi:10.1080/2162402X.2017.1393134. PMC5749653 29308325

[10] Legrand F, Driss V, Delbeke M, Loiseau S, Hermann E, Dombrowicz D, Capron M. Human eosinophils exert TNF-α and granzyme A-mediated tumoricidal activity toward colon carcinoma cells. J Immunol. 2010;185(12):7443–7451. doi:10.4049/jimmunol.1000446. 21068403

[11] Rosenberg HF, Dyer KD, Foster PS. Eosinophils: changing perspectives in health and disease. Nat Rev Immunol. 2013;13(1):9–22. doi:10.1038/nri3341. 23154224 PMC4357492

[12] Carretero R, Sektioglu IM, Garbi N, Salgado OC, Beckhove P, Hämmerling GJ. Eosinophils orchestrate cancer rejection by normalizing tumor vessels and enhancing infiltration of CD8+ T cells. Nat Immunol. 2015;16(6):609–617. doi:10.1038/ni.3159. 25915731

[13] Andreone S, Spadaro F, Buccione C, Mancini J, Tinari A, Sestili P, Gambardella AR, Lucarini V, Ziccheddu G, Parolini I, et al. IL-33 promotes CD11b/CD18-mediated adhesion of eosinophils to cancer cells and synapse-polarized degranulation leading to tumor cell killing. Cancers. 2019;11(11):1664. doi:10.3390/cancers11111664. PMC6895824 31717819

[14] Liew FY, Girard J-P, Turnquist HR. Interleukin-33 in health and disease. Nat Rev Immunol. 2016;16(11):676–689. doi:10.1038/nri.2016.95. 27640624

[15] Kim JY, Lim S-C, Kim G, Yun HJ, Ahn S-G, Choi HS. Interleukin-33/ST2 axis promotes epithelial cell transformation and breast tumorigenesis via upregulation of COT activity. Oncogene. 2015;34(38):4928–4938. doi:10.1038/onc.2014.418. 25531326

[16] Afferni C, Buccione C, Andreone S, Galdiero MR, Varricchi G, Marone G, Mattei F, Schiavoni G. The pleiotropic immunomodulatory functions of IL-33 and its implications in tumor immunity. Front Immunol. 2018;9:2601. doi:10.3389/fimmu.2018.02601. 30483263 PMC6242976

[17] Li Y, Shi J, Qi S, Zhang J, Peng D, Chen Z, Wang G, Wang Z, Wang L. IL-33 facilitates proliferation of colorectal cancer dependent on COX2/PGE2. J Exp Clin Cancer Res. 2018:37. doi:10.1186/s13046-018-0839-7. 30119635 PMC6098640

[18] Zhou Y, Ji Y, Wang H, Zhang H, Zhou H. IL-33 promotes the development of colorectal cancer through inducing tumor-infiltrating ST2L+ regulatory T cells in mice. Technol Cancer Res Treat. 2018;17:153303381878009. doi:10.1177/1533033818780091. PMC6048617 29950152

[19] He Z, Chen L, Souto FO, Canasto-Chibuque C, Bongers G, Deshpande M, Harpaz N, Ko HM, Kelley K, Furtado GC, et al. Epithelial-derived IL-33 promotes intestinal tumorigenesis in Apc Min/+ mice. Sci Rep. 2017;7(1):5520. doi:10.1038/s41598-017-05716-z. 28710436 PMC5511216

[20] Fang M, Li Y, Huang K, Qi S, Zhang J, Zgodzinski W, Majewski M, Wallner G, Gozdz S, Macek P, et al. IL33 promotes colon cancer cell stemness via JNK activation and macrophage recruitment. Cancer Res. 2017;77(10):2735–2745. doi:10.1158/0008-5472.CAN-16-1602. 28249897 PMC5760170

[21] Maywald RL, Doerner SK, Pastorelli L, De Salvo C, Benton SM, Dawson EP, Lanza DG, Berger NA, Markowitz SD, Lenz H-J, et al. IL-33 activates tumor stroma to promote intestinal polyposis. Proc Natl Acad Sci U S A. 2015;112(19):E2487–2496. doi:10.1073/pnas.1422445112. 25918379 PMC4434739

[22] Mertz KD, Mager LF, Wasmer M-H, Thiesler T, Koelzer VH, Ruzzante G, Joller S, Murdoch JR, Brümmendorf T, Genitsch V, et al. The IL-33/ST2 pathway contributes to intestinal tumorigenesis in humans and mice. Oncoimmunology. 2015;5:1. doi:10.1080/2162402X.2015.1062966. PMC4760343 26942077

[23] Zhang Y, Davis C, Shah S, Hughes D, Ryan JC, Altomare D, Peña MMO. IL-33 promotes growth and liver metastasis of colorectal cancer in mice by remodeling the tumor microenvironment and inducing angiogenesis. Mol Carcinog. 2017;56(1):272–287. doi:10.1002/mc.22491. 27120577 PMC5630136

[24] Liu X, Zhu L, Lu X, Bian H, Wu X, Yang W, Qin Q. IL-33/ST2 pathway contributes to metastasis of human colorectal cancer. Biochem Biophys Res Commun. 2014;453(3):486–492. doi:10.1016/j.bbrc.2014.09.106. 25280997

[25] Lucarini V, Ziccheddu G, Macchia I, La Sorsa V, Peschiaroli F, Buccione C, Sistigu A, Sanchez M, Andreone S, D’Urso MT, et al. IL-33 restricts tumor growth and inhibits pulmonary metastasis in melanoma-bearing mice through eosinophils. Oncoimmunology. 2017;6(6):e1317420. doi:10.1080/2162402X.2017.1317420. 28680750 PMC5486175

[26] Eissmann MF, Dijkstra C, Wouters MA, Baloyan D, Mouradov D, Nguyen PM, Davalos-Salas M, Putoczki TL, Sieber OM, Mariadason JM, et al. Interleukin 33 signaling restrains sporadic colon cancer in an interferon-γ-dependent manner. Cancer Immunol Res. 2018;6(4):409–421. doi:10.1158/2326-6066.CIR-17-0218. 29463593

[27] O’Donnell C, Mahmoud A, Keane J, Murphy C, White D, Carey S, O’Riordain M, Bennett MW, Brint E, Houston A. An antitumorigenic role for the IL-33 receptor, ST2L, in colon cancer. Br J Cancer. 2016;114(1):37–43. doi:10.1038/bjc.2015.433. 26679377 PMC4716545

[28] Komai-Koma M, Wang E, Kurowska-Stolarska M, Li D, McSharry C, Xu D. Interleukin-33 promoting Th1 lymphocyte differentiation dependents on IL-12. Immunobiology. 2016;221(3):412–417. doi:10.1016/j.imbio.2015.11.013. 26688508 PMC4731778

[29] Tosolini M, Kirilovsky A, Mlecnik B, Fredriksen T, Mauger S, Bindea G, Berger A, Bruneval P, Fridman W-H, Pagès F, et al. Clinical impact of different classes of infiltrating T cytotoxic and helper cells (Th1, Th2, Treg, Th17) in patients with colorectal cancer. Cancer Res. 2011;71(4):1263–1271. doi:10.1158/0008-5472.CAN-10-2907. 21303976

[30] Villarreal DO, Wise MC, Walters JN, Reuschel EL, Choi MJ, Obeng-Adjei N, Yan J, Morrow MP, Weiner DB. Alarmin IL-33 acts as an immunoadjuvant to enhance antigen-specific tumor immunity. Cancer Res. 2014;74(6):1789–1800. doi:10.1158/0008-5472.CAN-13-2729. 24448242 PMC4130175

[31] Lu B, Yang M, Wang Q. Interleukin-33 in tumorigenesis, tumor immune evasion, and cancer immunotherapy. J Mol Med. 2016;94(5):535–543. doi:10.1007/s00109-016-1397-0. 26922618

[32] Larsen KM, Minaya MK, Vaish V, Peña MMO. The role of IL-33/ST2 pathway in tumorigenesis. Int J Mol Sci. 2018;19(9):2676. doi:10.3390/ijms19092676. PMC6164146 30205617

[33] Hasenoehrl C, Feuersinger D, Sturm EM, Bärnthaler T, Heitzer E, Graf R, Grill M, Pichler M, Beck S, Butcher L, et al. G protein-coupled receptor GPR55 promotes colorectal cancer and has opposing effects to cannabinoid receptor 1. Int J Cancer. 2018;142(1):121–132. doi:10.1002/ijc.31030. 28875496 PMC5679368

[34] Busch SE, Hanke ML, Kargl J, Metz HE, MacPherson D, Houghton AM. Lung cancer subtypes generate unique immune responses. J.I. 2016;197(11):4493–4503. doi:10.4049/jimmunol.1600576. PMC5116260 27799309

[35] Reichman H, Rozenberg P, Munitz A. Mouse eosinophils: identification, isolation, and functional analysis. Curr Protoc Immunol. 2017;119(1):14.43.1–14.43.22. doi:10.1002/cpim.35. 29091265

[36] Dyer KD, Moser JM, Czapiga M, Siegel SJ, Percopo CM, Rosenberg HF. Functionally competent eosinophils differentiated ex vivo in high purity from normal mouse bone marrow. J Immunol. 2008;181(6):4004–4009. doi:10.4049/jimmunol.181.6.4004. 18768855 PMC2680436

[37] Frei RB, Luschnig P, Parzmair GP, Peinhaupt M, Schranz S, Fauland A, Wheelock CE, Heinemann A, Sturm EM. Cannabinoid receptor 2 augments eosinophil responsiveness and aggravates allergen-induced pulmonary inflammation in mice. Allergy. 2016;71(7):944–956. doi:10.1111/all.12858. 26850094 PMC5225803

[38] Knuplez E, Curcic S, Theiler A, Bärnthaler T, Trakaki A, Trieb M, Holzer M, Heinemann A, Zimmermann R, Sturm EM, et al. Lysophosphatidylcholines inhibit human eosinophil activation and suppress eosinophil migration in vivo. Biochim Biophys Acta (BBA). 2020;1865(7):158686. doi:10.1016/j.bbalip.2020.158686. 32171907

[39] Walker C, Rihs S, Braun RK, Betz S, Bruijnzeel PLB. Increased expression of CD11b and functional changes in eosinophils after migration across endothelial cell monolayers. J Immunol. 1993;150:4061–4071. 8097228

[40] Valencia HA, Loffredo LF, Misharin AV, Berdnikovs S. Phenotypic plasticity and targeting of Siglec-FhighCD11clow eosinophils to the airway in a murine model of asthma. Allergy. 2016;71(2):267–271. doi:10.1111/all.12776. 26414117

[41] Carmo LAS, Bonjour K, Ueki S, Neves JS, Liu L, Spencer LA, Dvorak AM, Weller PF, Melo RCN. CD63 is tightly associated with intracellular, secretory events chaperoning piecemeal degranulation and compound exocytosis in human eosinophils. J Leukoc Biol. 2016;100(2):391–401. doi:10.1189/jlb.3A1015-480R. 26965633 PMC6608091

[42] Palacios-Macapagal D, Connor J, Mustelin T, Ramalingam TR, Wynn TA, Davidson TS. Cutting edge: eosinophils undergo caspase-1–mediated pyroptosis in response to necrotic liver cells. J.I. 2017;199(3):847–853. doi:10.4049/jimmunol.1601162. 28652398

[43] Willebrand R, Voehringer D. IL-33-induced cytokine secretion and survival of mouse eosinophils is promoted by autocrine GM-CSF Ryffel B, editor. PLoS One. 2016;11(9):e0163751. doi:10.1371/journal.pone.0163751. 27690378 PMC5045177

[44] Stenfeldt A-L, Wennerås C. Danger signals derived from stressed and necrotic epithelial cells activate human eosinophils. Immunology. 2004;112(4):605–614. doi:10.1111/j.1365-2567.2004.01906.x. 15270732 PMC1782530

[45] Chen X, Lu K, Timko NJ, Weir DM, Zhu Z, Qin C, Mann JD, Bai Q, Xiao H, Nicholl MB, et al. IL‑33 notably inhibits the growth of colon cancer cells. Oncol Lett. 2018;16(1):769–774. doi:10.3892/ol.2018.8728. 29963144 PMC6019937

[46] Akimoto M, Maruyama R, Takamaru H, Ochiya T, Takenaga K. Soluble IL-33 receptor sST2 inhibits colorectal cancer malignant growth by modifying the tumour microenvironment. Nat Commun. 2016;7(1):1–15. doi:10.1038/ncomms13589. PMC5123057 27882929

[47] Johansson K, Malmhäll C, Ramos-Ramírez P, Rådinger M. Bone marrow type 2 innate lymphoid cells: a local source of interleukin-5 in interleukin-33-driven eosinophilia. Immunology. 2018;153(2):268–278. doi:10.1111/imm.12842. 28921511 PMC5765380

[48] Malik A, Sharma D, Zhu Q, Karki R, Guy CS, Vogel P, T-D K. IL-33 regulates the IgA-microbiota axis to restrain IL-1α-dependent colitis and tumorigenesis. J Clin Invest. 2016;126(12):4469–4481. doi:10.1172/JCI88625. 27775548 PMC5127671

[49] Jia S, Li W, Liu P, Xu LX. A role of eosinophils in mediating the anti-tumour effect of cryo-thermal treatment. Sci Rep. 2019;9(1):13214. doi:10.1038/s41598-019-49734-5. 31519961 PMC6744470

[50] Chu VT, Beller A, Rausch S, Strandmark J, Zänker M, Arbach O, Kruglov A, Berek C. Eosinophils promote generation and maintenance of immunoglobulin-A-expressing plasma cells and contribute to gut immune homeostasis. Immunity. 2014;40(4):582–593. doi:10.1016/j.immuni.2014.02.014. 24745334

[51] Hollande C, Boussier J, Ziai J, Nozawa T, Bondet V, Phung W, Lu B, Duffy D, Paradis V, Mallet V, et al. Inhibition of the dipeptidyl peptidase DPP4 (CD26) reveals IL-33-dependent eosinophil-mediated control of tumor growth. Nat Immunol. 2019;20(3):257. doi:10.1038/s41590-019-0321-5. 30778250

[52] Cherry WB, Yoon J, Bartemes KR, Iijima K, Kita H. A novel IL-1 family cytokine, IL-33, potently activates human eosinophils. J Allergy Clin Immunol. 2008;121(6):1484–1490. doi:10.1016/j.jaci.2008.04.005. 18539196 PMC2821937

[53] Bouffi C, Rochman M, Zust CB, Stucke EM, Kartashov A, Fulkerson PC, Barski A, Rothenberg ME. IL-33 markedly activates murine eosinophils by an NFκB-dependent mechanism differentially dependent upon an IL-4-driven autoinflammatory loop. J Immunol. 2013;191(8). doi:10.4049/jimmunol.1301465. PMC3807853 24043894

[54] Nazaroff CD, Rank MA, Guo J, Wright BL, Ochkur SI, Jacobsen EA. Eosinophil subtypes defined by distinct gene expression and function. J Allergy Clin Immunol. 2019;143(2):AB289. doi:10.1016/j.jaci.2018.12.884.

[55] Knuplez E, Krier‐Burris R, Cao Y, Marsche G, O’Sullivan J, Bochner BS. Frontline science: superior mouse eosinophil depletion in vivo targeting transgenic Siglec-8 instead of endogenous Siglec-F: mechanisms and pitfalls. J Leukoc Biol. 2020; doi:10.1002/JLB.3HI0120-381R. PMC7585130 32134149

[56] Andersson P, Yang Y, Hosaka K, Zhang Y, Fischer C, Braun H, Liu S, Yu G, Liu S, Beyaert R, et al. Molecular mechanisms of IL-33–mediated stromal interactions in cancer metastasis. JCI Insight. 2018;3(20). doi:10.1172/jci.insight.122375 PMC6237443 30333314

