# Supplementary material for: IL-33 reduces tumor growth in models of colorectal cancer with the help of eosinophils
Source: Oncoimmunology. 2020 Jun 16;9(1):1776059. doi: 10.1080/2162402X.2020.1776059 (PMC7458617; doi:10.1080/2162402X.2020.1776059)
Supplement: Supplemental Material [file KONI_A_1776059_SM1922.docx]

**Supplementary Fig. 1**


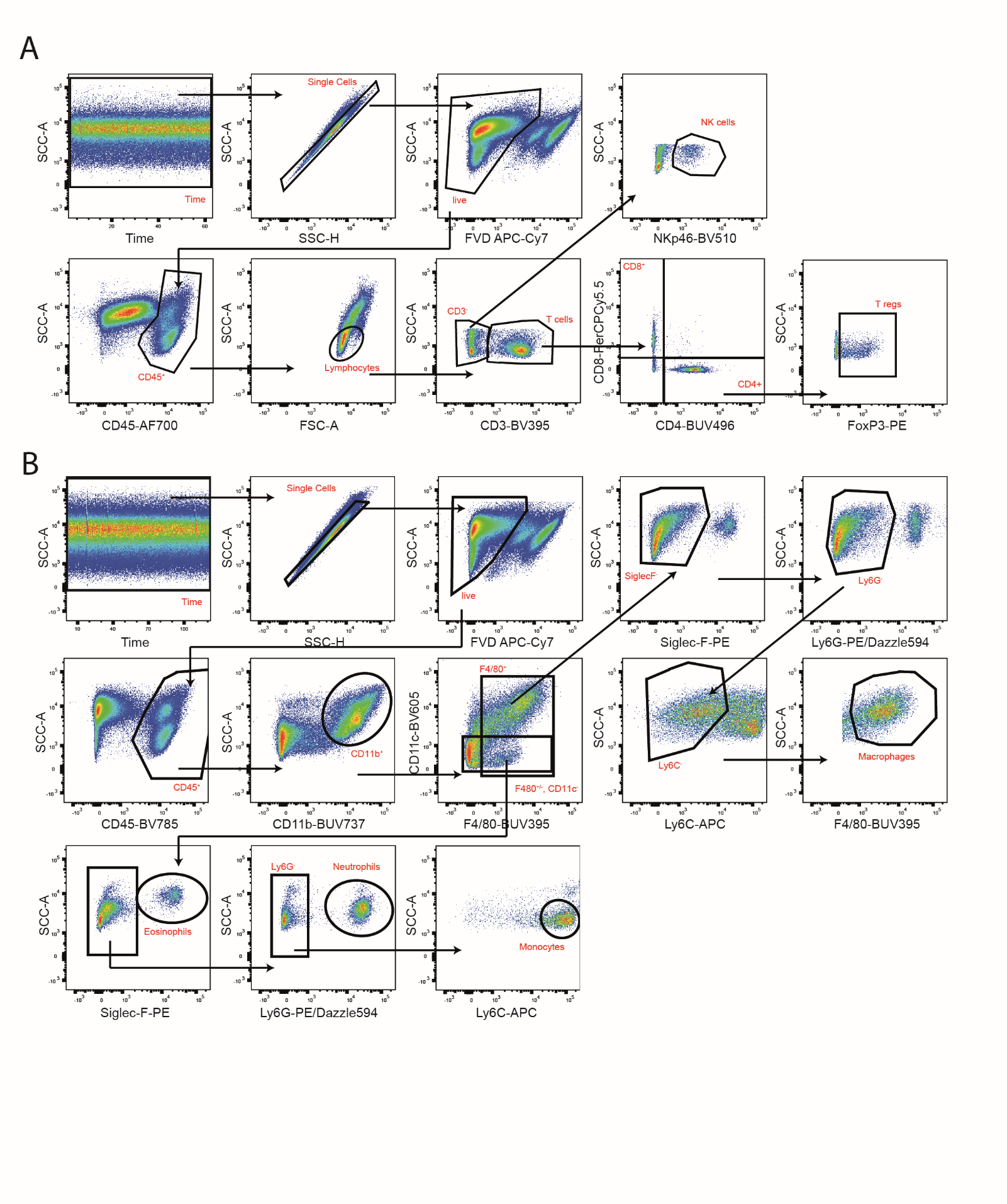


**Flow cytometry gating strategies**

Schematic presentation of the lymphoid (**A**) and myeloid (**B**) gating strategy for analysis of single cell suspensions of tumors. Tumor infiltrating leukocytes (CD45^+^) were pre-gated for time and single cells. Dead cells were excluded. (**A**) T cells were determined as CD45^+^/CD3^+^; NK cells as CD45^+^/CD3^-^/NKp46^+^; CD8^+^ T cells as CD45^+^/CD3^+^/CD8^+^; CD4^+^ T cells as CD45^+^/CD3^+^/CD4^+^ and regulatory T cells (T regs) as CD45^+^/CD3^+^/CD4^+^/FoxP3^+^.

(**B**) Eosinophils cells were identified as CD45^+^/CD11b^+^/CD11c^-^/F4/80^+^/Siglec-F^+^; neutrophils as CD45^+^/CD11b^+^/CD11c^-^/Ly6G^+^; monocytes as CD45^+^/CD11b^+^/CD11c^-^/Ly6G^-^/Ly6C^+^ and macrophages as CD45^+^/CD11b^+^/F4/80^+^/Siglec-F^-^/Ly6G^-^/Ly6C^-^

**Supplementary Fig. 2**

**
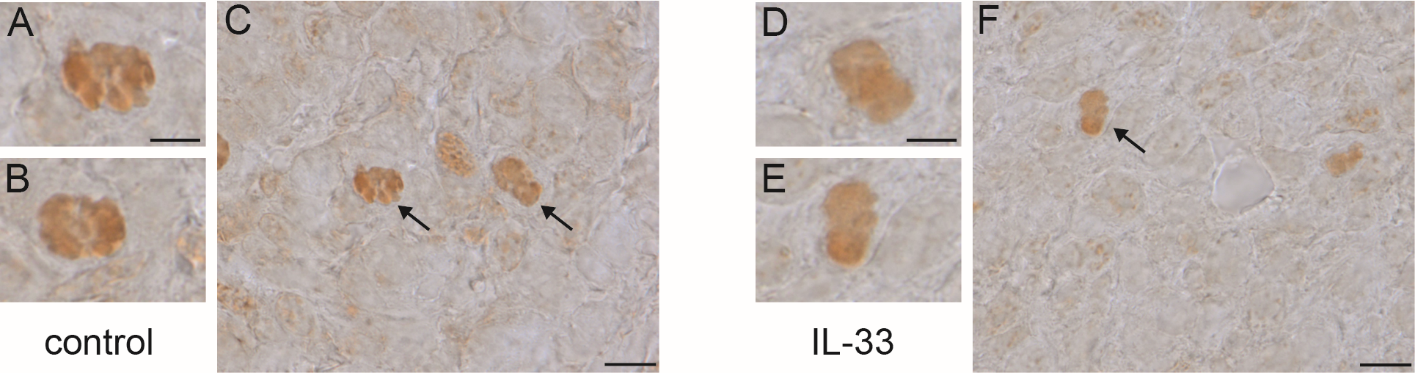
**

**Eosinophils in s.c. tumors of IL-33-treated mice appear less granulated.**

Immunohistochemical stainings with biotinylated mouse anti-EPX antibodies were performed to detect eosinophils in the tumor microenvironment of vehicle (control; **A-C**) and IL-33-treated (**D-F**) BALB/c mice (representative images from n=3/group). **A** and **B** show enlarged EPX-stained eosinophils with granules (calibration bar: 5µm). **C** shows eosinophils from vehicle-treated animals in a larger surrounding (arrows denote examples of EPX-stained eosinophils; calibration bar: 10µm). **D** and **E** show enlarged images of eosinophils from IL-33-treated mice with granules largely absent (calibration bar: 5µm). In **F**, arrow points at an eosinophil in a tumor section from an IL-33-treated mouse (calibration bar: 10µm)

**Supplementary Fig. 3**


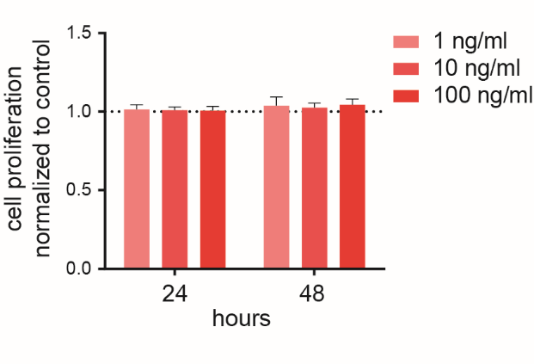


**CT26 cell proliferation upon IL-33 treatment**

The direct influence of IL-33 on the growth and viability of CT26 cells was investigated using a Cell titer 96 AQueous one solution assay performed according to the manufacturer’s instructions (Promega; G3581). In short, CT26 cells were seeded overnight in a 96-well plate. On the following day, cells were starved in media without FBS for 4 hrs. Afterwards, IL-33 was added in different concentrations (1-100 ng/ml) to the wells. Tumor cell proliferation was evaluated after 24 and 48 hrs on a xMark microplate spectrophotometer (Biorad, Hercules, CA, USA).

**Supplementary Fig. 4**


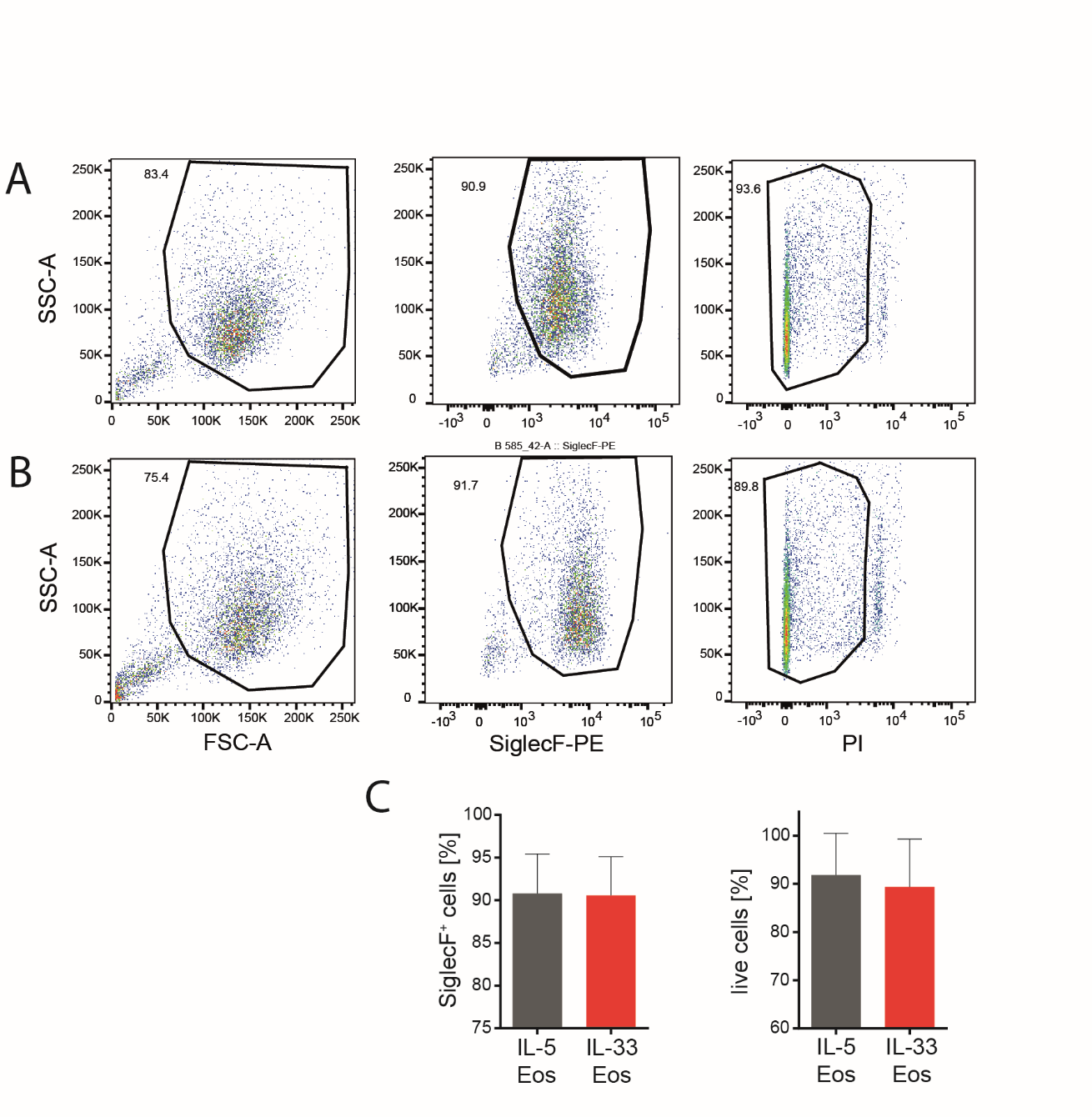


**Purity and viability of bone marrow-derived eosinophils (Eos)**

Eosinophils were differentiated from the bone marrow of BALB/c mice for 13 days and additionally treated with 100 ng/ml IL-33 for the last 20 hours. The purity (Siglec-F positive) and viability (propidium iodide [PI] negative) of (**A**) IL-5 Eos and (**B**) IL-33 Eos were about 90%. One representative plot is shown. (**C**) Quantitative analysis of purity and viability of IL-5 Eos and IL-33 Eos; n≤6.

**Supplementary Fig. 5**


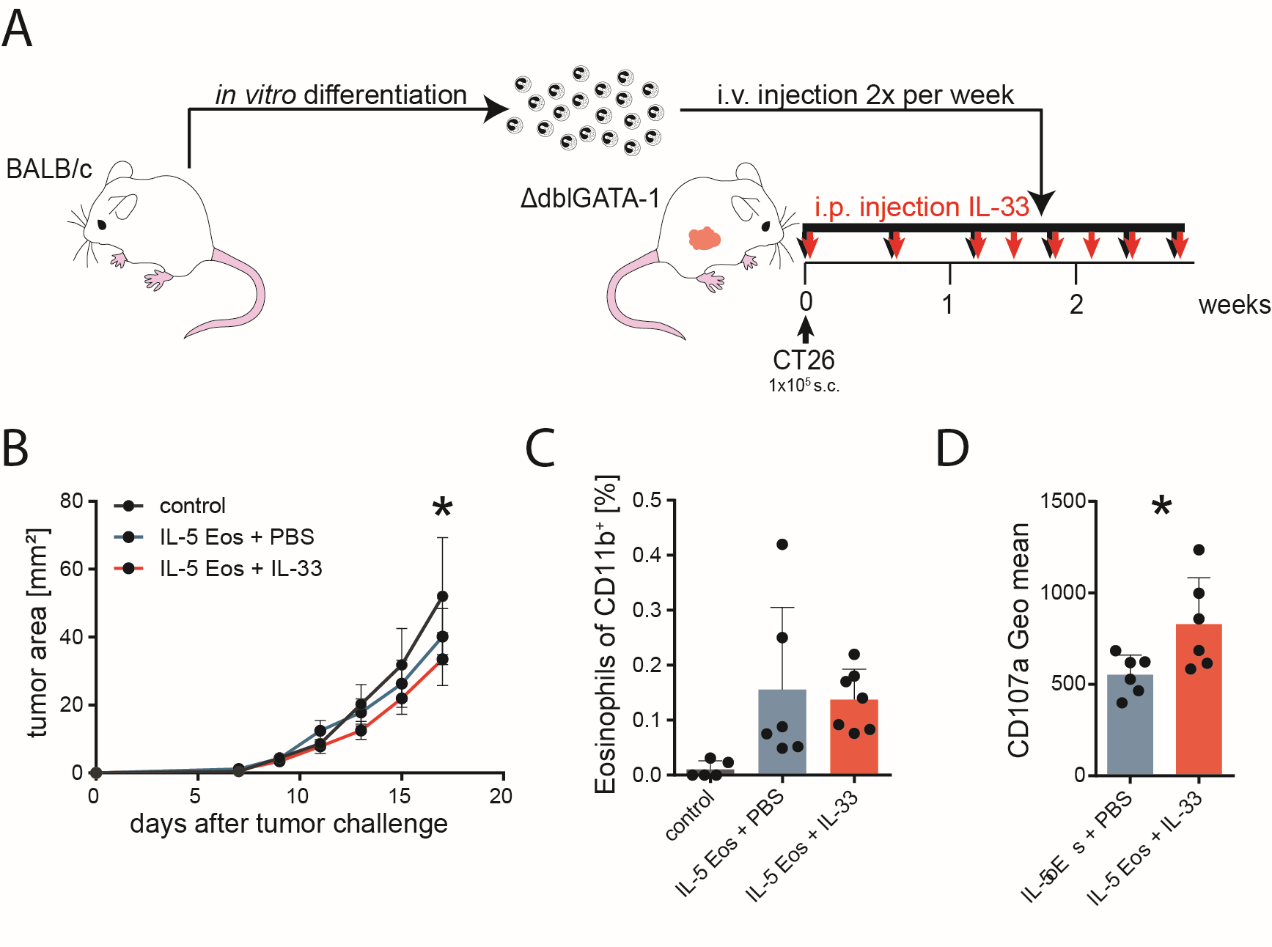


***In vivo* activation of adoptively-transferred IL-5 Eos by i.p. injections of IL-33**

(**A**) Schematic presentation of ∆dblGATA-1 mice with s.c. tumors that were repopulated with IL-5 Eos i.v. (twice weekly, black arrows) and additionally injected i.p. with 0.4 µg IL-33 (red arrows). Eosinophils for adoptive transfer were isolated from bone marrow of BALB/c mice and differentiated. ∆dblGATA-1 mice not receiving eosinophils served as controls (control). (**B**) Tumor growth was monitored during the course of the experiment; n≥6. Data indicate mean values ± SEM. *p<0.05 control vs. IL-5 Eos + IL-33 (**C**) Flow cytometry of tumor single cell suspensions was performed and infiltrating eosinophils were identified as % of CD11b^+^ cells. (**D**) Expression (Geo mean) of CD107a on infiltrated eosinophils was increased in mice that were additionally injected i.p. with IL-33. Statistical differences were assessed by using one-way ANOVA with Tukey’s multiple comparisons test and unpaired student’s *t*-test. *p<0.05.
